# Randomized-Controlled Trial Examining the Effect of Pelvic Floor Muscle Training in the Treatment of Stress Urinary Incontinence in Men after a Laparoscopic Radical Prostatectomy Pilot Study

**DOI:** 10.3390/jcm10132946

**Published:** 2021-06-30

**Authors:** Katarzyna Strojek, Magdalena Weber-Rajek, Agnieszka Strączyńska, Zuzanna Piekorz, Beata Pilarska, Piotr Jarzemski, Mariusz Kozakiewicz, Bartosz Brzoszczyk, Marcin Jarzemski, Hanna Styczyńska, Aleksander Goch, Agnieszka Radzimińska

**Affiliations:** 1 Department of Physiotherapy, Collegium Medicum in Bydgoszcz, Nicolaus Copernicus University in Torun, 85-067 Bydgoszcz, Poland; katarzyna.strojek@gmail.com (K.S.); magdawr69@gmail.com (M.W.-R.); zuzanna.piekorz@gmail.com (Z.P.); hanna.styczynska@cm.umk.pl (H.S.); katfizjoter@cm.umk.pl (A.G.); agnieszka.radziminska@gmail.com (A.R.); 2 Department of Urology, Collegium Medicum in Bydgoszcz, Nicolaus Copernicus University in Torun, 85-067 Bydgoszcz, Poland; beata.pilarska@cm.umk.pl (B.P.); piotr.jarzemski@cm.umk.pl (P.J.); 3 Clinic of Urology, Jan Biziel University Hospital No. 2 in Bydgoszcz, 85-168 Bydgoszcz, Poland; bartosz.brzoszczyk@gmail.com (B.B.); mjarzem@wp.pl (M.J.); 4 Department of Geriatrics, Collegium Medicum in Bydgoszcz, Nicolaus Copernicus University in Torun, 85-067 Bydgoszcz, Poland; markoz@cm.umk.pl

**Keywords:** pelvic floor muscle training, radical prostatectomy, urinary incontinence

## Abstract

Aim: The aim of this study was to assess the impact of pelvic floor muscle training (PMFT) in the treatment of stress urinary incontinence (SUI) in men after they received radical prostatectomy (RP). Methods: From November 2018 to September 2019, patients who underwent radical prostatectomy were assessed for eligibility. A total of 37 men were then randomly assigned to the experimental group (EG) and the control group (CG). The EG group received supervised exercise twice a week for 12 weeks, and the CG did not receive any intervention. To objectify the results obtained in both groups before and after the intervention, the authors assessed myostatin concentration. Moreover, the Expanded Prostate Cancer Index Composite (EPIC-26) was applied to assess the quality of life, and Beck’s Depression Inventory (BDI-II) was used to measure depression severity. Results: Study results demonstrated a statistically significant reduction of myostatin concentration in the EG following the treatment and no statistically significant differences in this parameter in the CG. In addition, a comparison of the EPIC-26 scores in the EG at the initial and final assessments revealed a statistically significant improvement in the quality of life in each domain. A comparison of the EPIC-26 scores in the CG at the initial and final assessments showed there is a statistically significant decline in quality of life in the “overall urinary problem” and “sexual” domain. A comparison of the BDI-II scores at the initial and final assessments showed a statistically significant decline in depressive symptoms in the EG and no statistically significant differences in the CG. Conclusions: PFMT is an effective treatment for urinary incontinence (UI) in men who received radical prostatectomy.

## 1. Introduction

Prostate cancer represents the second most common malignant tumor following lung cancer in men. A total of 1,276,106 people worldwide were diagnosed with this condition in 2018; the figure includes 358,989 deaths [1,2]. Frequency of prostate cancer worldwide correlates positively with age and affects nearly 60% of men over the age of 65 [3]. Approximately 81% of patients are diagnosed with early stage prostate cancer and so get the opportunity to receive effective treatment. One of the therapeutic options for patients in good condition is radical surgical removal of the prostate gland—radical prostatectomy. It offers 10-year survival rates in a group of patients with intermediate risk prostate cancer, as well as in selected patients with low- and high-risk prostate cancer [4]. The procedure involves removing the entire prostate with its capsule intact as well as seminal vesicles, followed by undertaking vesico-urethral anastomosis. Although advances in surgical techniques reduced postoperative complications after radical prostatectomy, this treatment method has two major side effects: urinary incontinence and erectile dysfunction. The prostate gland, which is located below the bladder, has the urethra pass through its center. The internal sphincter links the bladder with the urethra. The internal sphincter remains closed for the majority of the time and maintains continence. Another element involved in micturition control is the external sphincter, which is formed by the pelvic floor muscles. The removal of the prostatic segment of the urethra and its smooth muscle (internal sphincter) during radical prostatectomy may damage the striated urethral sphincter or its innervation. The smooth muscle of the bladder neck, as well as bladder contractility, may also be impacted by the surgery, leading to detrusor muscle overactivity [5]. Pelvic floor muscle training aims to activate pelvic floor muscles. Pelvic floor exercises were first described by A. H. Kegel in 1948 [6]. Properly and systematically performed exercises result in: better support for the pelvic organs, improvement of resting pressures in the urethra, extension of the functional length of the urethra, activation of the periurethral striated muscles, and as a result, an increase in the resting tension of the levator ani [7]. The method is recommended by the European Association of Urology in the treatment of urinary incontinence in men after radical prostatectomy [5,8,9,10]. Numerous methods, such as mechanomyography (MMG), electromyography (EMG), and ultrasound are applied to measure muscle activity [11,12,13]. In this study, we decided to assess varying results of biochemical parameters in response to the activation of pelvic floor muscles. We evaluated varying results in the myostatin concentration (Growth and Differentiation Factor 8: GDF-8) which a protein produced by using the skeletal muscle cells. The myostatin level increases in periods of skeletal muscle inactivity. However, therapeutic interventions may inhibit myostatin signaling [14,15]. Akita et al. [16] suggest in their paper that myostatin also inhibits the proliferation of satellite cells of skeletal muscles of the external sphincter. Therefore, inhibition of GDF-8 functions may be a useful strategy in treating stress urinary incontinence. This theory was confirmed in our previous research conducted on women with stress urinary incontinence, in which we observed a statistically significant reduction of myostatin concentration following various methods of activating pelvic floor muscles, such as pelvic floor muscle training and extracorporeal magnetic innervation (ExMI) [17,18]. The objective of this study was to evaluate the effectiveness of pelvic floor muscle training in patients who received radical prostatectomy and to examine its biochemical parameters. Nevertheless, guided by the biopsychosocial model of health, the authors also aimed to assess the quality of life of the study participants.

## 2. Methods

### 2.1. Study Design

Between November 2018 and September 2019, a total of 76 men with prostate cancer who received radical prostatectomy were enrolled into a randomized, controlled study. The study was conducted in accordance with the Declaration of Helsinki guidelines. Prior to the study, the authors obtained approval from the Bioethics Committee of the Collegium Medicum in Bydgoszcz, Nicolaus Copernicus University in Torun (KB: 562/2018).

Study inclusion criteria were as follows: patients after laparoscopic radical prostatectomy; stress urinary incontinence diagnosed by a urologist and based on urodynamic examination results; recent therapeutic interventions in pelvic floor performed within 6 months prior to the study (PFMT, magnetotherapy, electrostimulation, and biofeedback); no contradictions to the treatment; written consent to the study.

Study exclusion criteria were as follows: perineal surgery, robot-assisted surgery performed using the da Vinci system, or transurethral resection of the prostate (TURP); surgical and post-surgical complications disallowing early intervention with physiotherapy (damage to the external sphincter, urinary system infection, bladder neck stenosis); detrusor muscle overactivity; no incontinence following surgery; urinary incontinence before the surgery; active malignancy.

Stratified randomization was ensured by applying a simple subject allocation method. During the group allocation process, a blinded investigator picked a number from a computer-generated table. Each number was assigned to an envelope containing information on group allocation. Additionally, all patients provided written informed consent for the study. In the first stage of the study, 39 men were excluded (8 men did not meet the inclusion criteria and 31 men refused to participate). A total of 37 men were then randomly assigned to the experimental group (EG) and the control group (CG). Three men from the CG missed the final study visit. Consequently, 34 men completed the study (EG, *n* = 19; CG, *n* = 15). Moreover, we followed the methods of Weber-Rajek et al. 2019 and Radzimińska et al. 2018 [17,18].

**Figure 1:**
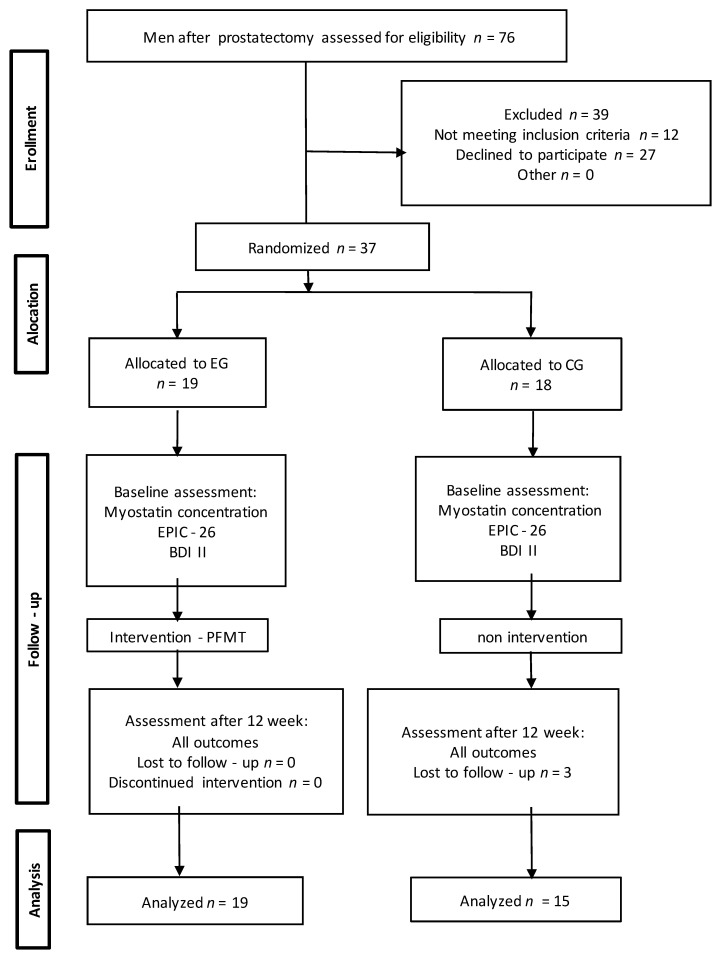
The study flow diagram. EG: experimental group; CG: control group; EPIC-26: Expanded Prostate Cancer Index Composite; BDI -II: Beck’s Depression Inventory; PFMT: pelvic floor muscle training.

The RCT reporting quality was improved using the CONSORT statement (Consolidated Standards of Reporting Trials (Figure 1)) [19].

### 2.2. Measurements

The following parameters were evaluated in the EG and CG at the initial and final assessments:

1. Myostatin concentration

The study design followed the procedure for the determination of myostatin in human serum and plasma outlined in the Myostatin ELISA manual (Immundiagnostik AG, Bensheim, Germany; cat. no: K 1012). Six milliliters of blood were collected from each participant on an empty stomach into Vacuette tubes with EDTA anticoagulant. Afterwards, the collected samples were centrifuged at 3000 rpm for 15 min to obtain plasma, which was then pipetted into smaller samples of about 500 µL and frozen at −80 °C. Next, the researchers read the results using the BMG Labtech ELISA absorbance reader with a monochromator. Consequently, the assay allowed for quantitative determination of myostatin in EDTA plasma and serum samples. First, the researchers added a biotinylated myostatin tracer to the samples, controls, and standards. Next, they transferred and incubated aliquots of the treated preparations into microtiter plate wells coated with polyclonal anti-myostatin antibodies. Once the incubation phase commences, the free target antigen in the samples competes with the biotinylated myostatin tracer, and afterwards it binds with the polyclonal anti-myostatin antibodies immobilized on the microtiter plate wells. The researchers removed the unbound components at the washing step. During the second incubation step, each microtiter received a streptavidin-labeled peroxidase antibody, which binds to the biotinylated myostatin tracer. Once the unbound components were removed at the washing step, the researchers added peroxidase substrate tetramethylbenzidine (TMB). In the final step, the acidic stop solution terminated the enzymatic reaction and changed the sample color from blue to yellow. It is essential to note that the yellow color intensity is inversely proportional to the myostatin concentration. The sample color intensity was measured at 450 nm. The weaker photometric signal is the consequence of a high myostatin concentration, which affects the sample by lowering the concentration of the biotinylated myostatin tracer bound to the immobilized anti-myostatin antibodies [17,18].

2. EPIC-26

Health-related quality of life (HRQoL) issues of patients with prostate cancer are assessed using the 50-item Expanded Prostate Cancer Index Composite (EPIC) instrument, which extends the original 20-item University of California, Los Angeles Prostate Cancer Index (UCLA-PCI) with additional items and is designed to evaluate irritating symptoms, as well as impacts of hormonal therapy. EPIC-26 is a brief 26-item version of the original EPIC instrument, which was developed to enhance the tool’s utility. The EPIC-26 includes 26 items within the following five domains: incontinence subscale (four items), irritation/obstruction subscale (four items), bowel symptom (six items), sexual symptom (six items), vitality or hormonal symptom (five items), and overall urinary difficulties (one item). Better health-related quality of life is represented with higher scores [20].

3. BDI-II

Beck’s Depression Inventory is commonly used to measure depression severity in patients. This self-scored depression scale is widely used in studies on mental disorders. Moreover, the instrument may be used to rate the mood of oncological, urological, gynecological, and neurological patients. The BDI-II questionnaire consists of 21 items, self-scored by patients on a scale of 0–3, where 0 means no symptoms and 3 stands for severe symptoms. The score of 0–8 means no depression, 9–18 stands for moderate depression, and the score of 18 points or more is interpreted as severe depression [21].

### 2.3. Intervention

The EG received 24 individual sessions of physiotherapist-guided PFMT (twice a week over 3 months) two weeks following the surgery. The exercises were conducted in a hospital rehabilitation department. Prior to the therapeutic intervention, each study participant underwent postural correction. Afterwards, sacroiliac joints and sacro-lumbar joints were mobilized, and the participants were taught thoracic and abdominal respiration. Once the above-mentioned procedures were completed, the study participants began PFMT activating fast-twitch fibers and slow-twitch fibers with cocontraction of the transverse abdominal muscle. The study participants performed the exercises in standing, supine, and sitting positions. The number of exercise sets and the contraction time of pelvic floor muscle fibers were individually adjusted to each study participant and his functional activity.

### 2.4. Statistical Analyses

The analyses were conducted with R 4.02. statistical software (CIT). Due to the presence of ties, small and unequal sample sizes, and skewed distributions of the variables, we used nonparametric permutation tests implemented in package coin (CIT.), with *p* values approximated via a Monte Carlo simulation based on 10,000 samples. To compare the experimental and control groups, the Wilcoxon-Mann-Whitney (WMW) test was used, and to compare the change in parameter values before vs. after the treatment, the Wilcoxon signed-rank (WSR) test was used. The data distributions were summarized with the median (Me), interquartile range (IQR), skewness (Sk.), minimum (Min), and maximum (Max). For the paired tests, we also developed a summary of the distributions of within participant differences in parameter values. Effect sizes were evaluated with r statistic defined as r = Z/n, where Z is the test statistic, and n is the number of observations and pairs of observations for unpaired and paired tests, respectively. For each set of comparisons (i.e., before the treatment, after the treatment and treatment effects within the experimental and control groups) a Holm correction for multiple comparisons was used to control the family wise errors.

## 3. Results

Study population characteristics are listed in Table 1.

Table 2 presents a statistical analysis of variables measured in the EG and the CG at the initial assessment used to define homogeneity of the study groups.

We found that the groups did not differ significantly before the treatment in seven out of eight measured parameters. The experimental group had statistically significant and moderately higher average level of the myostatin concentration than the control group.

Table 3 shows a comparison of variables measured in the EG during the initial and final assessments.

We observed a statistically significant and large reduction of the parameter values after the treatment for the overall urinary difficulties, incontinence, irritation/obstruction, vitality symptoms and sexual symptoms subscales, BDI-II scores, and the myostatin concentration levels.

Table 4 shows a comparison of variables measured in the CG at the initial and final assessments.

We observed almost no significant changes in parameter values after the treatment within the control group. The only significant results were a large reduction in the values of the overall urinary difficulties and sexual symptoms scales.

Table 5 demonstrates a comparison of variables measured for the EG and the CG at the final assessment.

After the treatment, the groups differed in six out of eight measured parameters. The experimental group, as compared to the control group, had a substantially lower average level of urinary difficulties, as well as moderately lower average scores on the incontinence and irritation/obstruction scale and on the vitality or hormonal symptoms and bowel domain. Additionally, the experimental group showed moderately lower scores on the BDI-II scale than the control group.

## 4. Discussion

Urinary incontinence is a complication following RP which impairs most physical and psychosocial functioning of men affected by this condition [22,23]. In this study, we assessed the effectiveness of PFMT in men who received RP. Both physical functioning and health-related quality of life (HRQoL) were evaluated in order to objectify treatment outcomes. A statistically significant reduction of myostatin concentration was observed in the EG (*p* < 0.001) following PFMT, and no statistically significant differences in this parameter were observed in the CG (*p* = 0.339) at the final assessment. Similar results were obtained in our earlier research conducted on a group of women with stress urinary incontinence [17,18].

In addition, patients’ HRQoL was evaluated using the Expanded Prostate Cancer Index Composite (EPIC-26). A comparison of the results in the EG at the initial and final assessments revealed a statistically significant improvement in the quality of life in each EPIC-26 domain. Interestingly, a comparison of the results in the CG at the initial and final assessments showed there is a statistically significant decline in quality of life in the overall urinary problem and sexual domain. These findings confirm the theory that out of the two main complications following RP, UI is the one that reduces men’s quality of life the most.

Furthermore, among numerous comorbid conditions associated with UI, the literature on the subject identifies depression as the most debilitating mental health condition [24,25]. The study group reported mild depression severity which was measured using the Beck Depression Inventory II. The EG results at the initial assessment were: no depression in 13 patients, moderate depression in 5 patients, and severe depression in one patient. The CG results at initial assessment were: no depression in 12 patients and moderate depression in 3 patients. The EG results at final assessment were: no depression in 18 patients and moderate depression in one patient. Whereas the CG results at final assessment were: no depression in 11 patients and moderate depression in 4 patients. However, more importantly, a comparison of the BDI-II scores at the initial and final assessments showed a statistically significant decline in depressive symptoms in the EG and no statistically significant differences in the CG.

## 5. Conclusions

PFMT is an effective method of treating urinary incontinence in men who received RP. The authors demonstrated that there is a statistically significant reduction of myostatin concentration, which may be a marker of pelvic floor muscle function; however, further research is required. Furthermore, an improvement in the quality of life in the study group was reported. The study provides evidence for PFMT to be implemented as standard practice for UI in men after radical prostatectomy.

## 6. Limitations

The authors are aware of limitations of their study, which include the lack of assessment of long-term treatment outcomes and a rather small study group and small difference between groups at the baseline point. Therefore, the authors consider this research to be a pilot study and intend to explore this topic further.

## Figures and Tables

**Table 1:** Study population characteristics.

Characteristics	EG	CG
Patients (n)	19	15
Age (y)	61.4 ± 7.4	64.2 ± 4.5
BMI (kg/m^2^)	26.2 ± 3.0	26.8 ± 2.7
Preoperative PSA (ng/mL)	8.3 ± 3.9	8.2 ± 3.6
T stage		
pT2b	2 (10.6)	2 (13.4)
pT2c	7 (36.8)	7 (46.6)
pT2a	4 (21)	1 (6.6)
pT2b	3 (15.8)	2 (13.4)
pT2c	3 (15.8)	3 (20)
Gleason score	6.7 ± 0.5	6.5 ± 0.5
Duration of catheterization (d)	8.6 ± 1.8	9.9 ± 1.9

EG: experimental group; CG: control group; BMI: Body Mass Index; PSA: Prostate Specific Antigen; T stage: the stage of prostate cancer—TNM system (T: tumor; N: nodes; M: metastasized); Data presented as mean ± SD or numbers with percentages in parentheses.

**Table 2:** Comparative analysis of all measured variables for the EG and the CG at the initial assessment.

Parameter	Group	Med	IQR	Min	Max	Sk.	Z	*p*	r
EPIC-26 (overall urinary difficulties)	CG	2	2.5	1	5	0.64	−0.14	1	0.02
	EG	2	3	1	5	0.4			
EPIC-26 (incontinence subscale)	CG	9	0	7	10	−1.24	−0.14	1	0.02
	EG	9	0	6	10	−2.05			
EPIC-26 (irritation/ obstruction subscale)	CG	4	4	0	9	0.38	0.6	1	0.1
	EG	2	5	0	10	0.73			
EPIC-26 (vitality or hormonal symptom)	CG	2	3.5	0	8	0.71	1.38	1	0.24
	EG	1	4	0	7	0.7			
EPIC-26 (bowel domain)	CG	4	5.5	1	8	0.17	2.24	0.2	0.38
	EG	1	0	0	14	2.46			
EPIC-26 (sexual symptom)	CG	19	2	11	24	−0.54	−1.93	0.371	0.33
	EG	22	6	10	25	−1.1			
BDI-II	CG	5	4	0	17	1.1	−0.51	1	0.09
	EG	5	8	0	20	0.77			
myostatin concentration	CG	91.91	24.33	65.28	169.76	0.94	−3.83	<0.001	0.66
	EG	146.02	49.28	99.38	188.62	−0.14			

EG: experimental group; CG: control group; Min: minimum; Max: maximum; Med: median; IQR: interquartile range; Sk: skewness, Z: Z statistic; r: r statistic; *p*: significance level.

**Table 3:** Comparative analysis of variables measured for the EG at the initial and final assessments.

Parameter	Measur.	Med	IQR	Min	Max	Sk.	Z	*p*	r
EPIC-26 (overall urinary difficulties)	B	2	3	1	5	0.4			
	A	1	0.5	0	4	1			
	A-B	−1	1.5	−4	0	−0.98	3.5	0.0007	0.8
EPIC-26 (incontinence subscale)	B	9	0	6	10	−2.05			
	A	6	3	0	9	−1.05			
	A-B	−2	3	−9	0	−1.42	3.47	0.0012	0.8
EPIC-26 (irritation/obstruction subscale)	B	2	5	0	10	0.73			
	A	1	1.5	0	9	2.57			
	A-B	−1	3.5	−9	1	−1.23	3.1	0.0033	0.71
EPIC-26 (vitality or hormonal symptom)	B	1	4	0	7	0.7			
	A	0	1	0	3	1.45			
	A-B	−1	2.5	−6	0	−1.16	3.11	0.0042	0.71
EPIC-26 (bowel domain)	B	1	0	0	14	2.46			
	A	1	1	0	9	2.22			
	A-B	0	1	−13	8	−1.21	1.8	0.0908	0.41
EPIC-26 (sexual symptom)	B	22	6	10	25	−1.1			
	A	10	1	0	22	0.9			
	A-B	−12	9	−23	−2	−0.11	3.83	<0.001	0.88
BDI-II	B	5	8	0	20	0.77			
	A	1	2.5	0	12	1.95			
	A-B	−4	4	−19	0	−1.56	3.65	0.0012	0.84
myostatin concentration	B	146.02	49.28	99.38	188.62	−0.14			
	A	118.25	43.32	63.35	171.53	−0.07			
	A-B	−20.83	22.05	−110.39	−0.55	−1.83	3.82	<0.001	0.88

Min: minimum; Max: maximum; Med: median; IQR: interquartile range; Sk: skewness, Z: Z statistic; r: r statistic; *p*: significance level.

**Table 4:** Comparative analysis of variables measured for the CG at the initial and final assessments.

Parameter	Measur.	Med	IQR	Min	Max	Sk.	Z	*p*	r
EPIC-26 (overall urinary difficulties)	B	2	2.5	1	5	0.64			
	A	5	1	3	5	−0.84			
	A-B	3	2	−2	4	−0.85	−3.01	0.0096	0.78
EPIC-26 (incontinence subscale)	B	9	0	7	10	−1.24			
	A	9	1	7	10	−0.37			
	A-B	0	1	−2	2	0	0	1	0
EPIC-26 (irritation/obstruction subscale)	B	4	4	0	9	0.38			
	A	4	5.5	0	9	−0.05			
	A-B	1	3	−7	8	0.09	−1.27	0.7492	0.33
EPIC-26 (vitality or hormonal symptom)	B	2	3.5	0	8	0.71			
	A	5	5	0	15	0.96			
	A-B	1	7.5	−4	13	0.59	−1.34	0.7492	0.35
EPIC-26 (bowel domain)	B	4	5.5	1	8	0.17			
	A	6	4.5	1	11	−0.08			
	A-B	1	3	−2	6	0.44	−2.07	0.2884	0.53
EPIC-26 (sexual symptom)	B	19	2	11	24	−0.54			
	A	10	1	6	15	0.56			
	A-B	−9	2.5	−14	4	1.65	3.36	0.0009	0.87
IPSS	B	11	8	3	28	0.97			
	A	18	14	7	28	−0.05			
	A-B	2	13.5	−6	23	0.76	−1.76	0.402	0.46
BDI-II	B	5	4	0	17	1.1			
	A	5	6.5	0	15	0.53			
	A-B	1	4.5	−13	8	−0.86	−0.6	1	0.15
myostatin concentration	B	91.91	24.33	65.28	169.76	0.94			
	A	91.47	33.2	45.98	162.71	0.34			
	A-B	−5.21	7.71	−19.3	15.6	0.19	1.93	0.339	0.5

Min: minimum; Max: maximum; Med: median; IQR: interquartile range; Sk: skewness, Z: Z statistic; r: r statistic; *p*: significance level.

**Table 5:** Comparative analysis of all measured variables for the EG and the CG at the final assessment.

Parameter	Group	Med	IQR	Min	Max	Sk.	Z	*p*	r
EPIC-26 (overall urinary difficulties)	CG	5	1	3	5	−0.84	4.65	<0.001	0.8
	EG	1	0.5	0	4	1			
EPIC-26 (incontinence subscale)	CG	9	1	7	10	−0.37	3.05	0.008	0.52
	EG	6	3	0	9	−1.05			
EPIC-26 (irritation/ obstruction subscale)	CG	4	5.5	0	9	−0.05	3.54	<0.001	0.61
	EG	1	1.5	0	9	2.57			
EPIC-26 (vitality or hormonal symptom)	CG	5	5	0	15	0.96	3.75	<0.001	0.64
	EG	0	1	0	3	1.45			
EPIC-26 (bowel domain)	CG	6	4.5	1	11	−0.08	3.54	<0.001	0.61
	EG	1	1	0	9	2.22			
EPIC-26 (sexual symptom)	CG	10	1	6	15	0.56	−0.27	0.784	−0.05
	EG	10	1	0	22	0.9			
	EG	1	2.5	0	8	1.18			
BDI-II	CG	5	6.5	0	15	0.53	2.58	0.03	0.44
	EG	1	2.5	0	12	1.95			
myostatin concentration	CG	91.47	33.2	45.98	162.71	0.34	−1.68	0.184	−0.29
	EG	118.25	43.32	63.35	171.53	−0.07			

EG: experimental group; CG: control group; Min: minimum; Max: maximum; Med: median; IQR: interquartile range; Sk: skewness, Z: Z statistic; r: r statistic; *p*: significance level.

## Data Availability

The data sets generated during and/or analyzed during the current study are available from the corresponding author on reasonable request.

## References

[1] Bray F., Ferlay J., Soerjomataram I., Siegel R.L., Torre L.A., Jemal A. (2018). Global cancer statistics 2018: GLOBOCAN estimates of incidence and mortality worldwide for 36 cancers in 185 countries. CA Cancer J. Clin..

[2] Ferlay J., Colombet M., Soerjomataram I., Mathers C., Parkin D.M., Pineros M., Znaor A., Bray F. (2019). Estimating the global cancer incidence and mortality in 2018: GLOBOCAN sources and methods. Int. J. Cancer.

[3] Rawla P. (2019). Epidemiology of Prostate Cancer. World J. Oncol..

[4] Mottet N., Van den Bergh R.C.N., Briers E., Cornford M., De Santis S., Fanti S., Gillessen J., Grummet A.M., Henry T.B., Lam M.D. (2019). European Association of Urology Guidelines 2019.

[5] Hodges P., Stafford R., Coughlin G.D., Kasza J., Ashton-Miller J., Cameron A.P., Connelly L., Hall L. (2019). Efficacy of a personalised pelvic floor muscle training programme on urinary incontinence after radical prostatectomy (MaTchUP): Protocol for a randomised controlled trial. BMJ Open.

[6] Kegel A.H. (1948). Progressive resistance exercise in the functional restoration of the perineal muscles. Am. J. Obstet. Gynecol..

[7] Bø K., Talseth T., Holme I. (1999). Single blind, randomised controlled trial of pelvic floor exercises, electrical stimulation, vaginal cones, and no treatment in management of genuine stress incontinence in women. BMJ.

[8] Schröder A., Abrams P., Andersson K.E. (2010). EAU guidelines on urinary incontinence. Eur. Assoc. Urol..

[9] Macdonald R., Fink H.A., Huckabay C., Monga M., Wilt T.J. (2007). Pelvic floor muscle training to improve urinary incontinence after radical prostatectomy: A systematic review of effectiveness. BJU Int..

[10] Kannan P., Winser S.J., Fung B., Cheing G. (2018). Effectiveness of Pelvic Floor Muscle Training Alone and in Combination with Biofeedback, Electrical Stimulation, or Both Compared to Control for Urinary Incontinence in Men Following Prostatectomy: Systematic Review and Meta-Analysis. Phys. Ther..

[11] Talib I., Sundaraj K., Lam C.K., Sundaraj S. (2018). A systematic review of muscle activity assessment of the biceps brachii muscle us-ing mechanomyography. J. Musculoskelet. Neuronal Interact..

[12] Stafford R.E., Coughlin G., Lutton N.J., Hodges P.W. (2015). Validity of Estimation of Pelvic Floor Muscle Activity from Transperineal Ultra-sound Imaging in Men. PLoS ONE.

[13] Neumann P., Fuller A., Sutherland P. (2015). Verbal pelvic floor muscle instructions pre-prostate surgery assessed by transperineal ultrasound: Do men get it?. Aust. N. Z. Cont. J..

[14] Roberts S.B., Goetz F.W. (2001). Differential skeletal muscle expression of myostatin across teleost species, and the isolation of multiple myostatin isoforms. FEBS Lett..

[15] Shin S., Song Y., Ahn J., Kim E., Chen P., Yang S., Suh Y., Lee K. (2015). A novel mechanism of myostatin regulation by its alternative splicing variant during myogenesis in avian species. Am. J. Physiol. Physiol..

[16] Akita Y., Sumino Y., Mori K.-I., Nomura T., Sato F., Mimata H. (2012). Myostatin inhibits proliferation of human urethral rhabdosphincter satellite cells. Int. J. Urol..

[17] Weber-Rajek M., Radzimińska A., Strączyńska A., Podhorecka M., Kozakiewicz M., Perkowski R., Jarzemski P., Kędziora-Kornatowska K., Goch A. (2018). A randomized-controlled trial pilot study examining the effect of extracorporeal magnetic innervation in the treatment of stress urinary incontinence in women. Clin. Interv. Aging.

[18] Radzimińska A., Weber-Rajek M., Strączyńska A., Podhorecka M., Kozakiewicz M., Kędziora-Kornatowska K., Goch A. (2018). The impact of pelvic floor muscle training on the myostatin concentration and severity of urinary incontinence in elderly women with stress urinary incontinence—A pilot study. Clin. Interv. Aging.

[19] Diallo S., Cour F., Josephson A., Vidart A., Botto H., Lebret T., Bonan B. (2012). Evaluating Single-incision Slings in Female Stress Urinary Incontinence: The Usefulness of the CONSORT Statement Criteria. Urology.

[20] Szymanski K.M., Wei J.T., Dunn R.L., Sanda M.G. (2010). Development and Validation of an Abbreviated Version of the Expanded Prostate Cancer Index Composite Instrument for Measuring Health-related Quality of Life Among Prostate Cancer Survivors. Urology.

[21] Beck A.T., Steer R.A., Brown G.K. (1996). BDI-II. Beck Depression Inventory. Manual.

[22] Holze S., Mende M., Healy K.V., Koehler N., Gansera L., Truss M.C., Rebmann U., Degener S., Stolzenburg J.-U. (2019). Comparison of various continence definitions in a large group of patients undergoing radical prostatectomy: A multicentre, prospective study. BMC Urol..

[23] Carrier J., Edwards D., Harden J. (2018). Men’s perceptions of the impact of the physical consequences of a radical prostatectomy on their quality of life. JBI Database Syst. Rev. Implement. Rep..

[24] Boeri L., Capogrosso P., Ventimiglia E., Cazzaniga W., Pederzoli F., Gandaglia G., Finocchio N., Dehò F., Briganti A., Montanari E. (2018). Depressive Symptoms and Low Sexual Desire after Radical Prostatectomy: Early and Long-Term Outcomes in a Real-Life Setting. J. Urol..

[25] Felde G., Ebbesen M.H., Hunskaar S. (2017). Anxiety and depression associated with urinary incontinence. A 10-year follow-up study from the Norwegian HUNT study (EPINCONT). Neurourol. Urodyn..

